# An Experimental Model of Acute Pulmonary Damage Induced by the Phospholipase A_2_-Rich Venom of the Snake *Pseudechis papuanus*

**DOI:** 10.3390/toxins17060302

**Published:** 2025-06-12

**Authors:** Daniela Solano, Alexandra Rucavado, Teresa Escalante, Edith Bastos Gandra Tavares, Suellen Karoline Moreira Bezerra, Clarice Rosa Olivo, Edna Aparecida Leick, Julio Alejandro Rojas Moscoso, Lourdes Dias, Iolanda de Fátima Lopes Calvo Tibério, Stephen Hyslop, José María Gutiérrez

**Affiliations:** 1 Instituto Clodomiro Picado, Facultad de Microbiología, Universidad de Costa Rica, San José 11501, Costa Rica; dsolano27@gmail.com (D.S.); alexandra.rucavado@ucr.ac.cr (A.R.); teresa.escalante@ucr.ac.cr (T.E.); 2 Departamento de Farmacologia, Faculdade de Ciências Médicas, Universidade Estadual de Campinas (UNICAMP), Rua Vital Brazil, 80, Cidade Universitária Zeferino Vaz, Campinas 13083-888, SP, Brazil; edith_bastos@yahoo.com.br (E.B.G.T.); caipisco@hotmail.com (J.A.R.M.); phdlourdes@gmail.com (L.D.); hyslop@unicamp.br (S.H.); 3 Faculdade de Medicina, Universidade de São Paulo (USP), Avenida Dr. Arnaldo, 455, Cerqueira César, São Paulo 01246-903, SP, Brazil; suellen.karoline93@gmail.com (S.K.M.B.); clariceolivo@gmail.com (C.R.O.); leick51@yahoo.com.br (E.A.L.); iocalvo@uol.com.br (I.d.F.L.C.T.)

**Keywords:** acute pulmonary damage, nitric oxide, phospholipase A_2_, *Pseudechis papuanus* venom, pulmonary edema

## Abstract

An experimental model of acute pulmonary damage was developed based on the intravenous injection of the phospholipase A_2_ (PLA_2_)-rich venom of *Pseudechis papuanus* (Papuan black snake) in mice. Venom caused pulmonary edema, with the accumulation of a protein-rich exudate, as observed histologically and by analysis of bronchoalveolar lavage fluid (BALF). In parallel, venom induced an increase in all of the pulmonary mechanical parameters evaluated, without causing major effects in terms of tracheal and bronchial reactivity. These effects were abrogated by incubating the venom with the PLA_2_ inhibitor varespladib, indicating that this hydrolytic enzyme is responsible for these alterations. The venom was cytotoxic to endothelial cells in culture, hydrolyzed phospholipids of a pulmonary surfactant, and reduced the activity of angiotensin-converting enzyme in the lungs. The pretreatment of mice with the nitric oxide synthase inhibitor L-NAME reduced the protein concentration in the BALF, whereas no effect was observed when mice were pretreated with inhibitors of cyclooxygenase (COX), tumor necrosis factor-α (TNF-α), bradykinin, or neutrophils. Based on these findings, it is proposed that the rapid pathological effect of this venom in the lungs is mediated by (a) the direct cytotoxicity of venom PLA_2_ on cells of the capillary–alveolar barrier, (b) the degradation of surfactant factor by PLA_2_, (c) the deleterious action of nitric oxide in pulmonary tissue, and (d) the cytotoxic action of free hemoglobin that accumulates in the lungs as a consequence of venom-induced intravascular hemolysis. Our findings offer clues on the mechanisms of pathophysiological alterations induced by PLA_2_s in a variety of pulmonary diseases, including acute respiratory distress syndrome (ARDS).

## 1. Introduction

Snakebite envenoming is a neglected tropical disease that exerts a significant public health burden, especially in sub-Saharan Africa, Asia, Latin America and parts of Oceania, including Papua New Guinea [1]. These envenomings are associated with multiple pathophysiological effects, resulting from the direct toxic action of venom components in several systems, as well as from the onset of endogenous inflammatory processes [1,2]. Snake venoms and toxins constitute useful tools for studying pathological and pathophysiological processes, such as skeletal muscle degeneration and regeneration [3,4,5] and inflammation [6], among others. However, the study of pulmonary alterations induced by snake venoms and toxins to understand lung pathophysiology has received less attention.

Pulmonary hemorrhage has been described in clinical envenomings by various species of viperid snakes [7,8,9]. These contain hemorrhagic metalloproteinases that disrupt the integrity of microvessels [10] and procoagulant enzymes that cause venom-induced consumption coagulopathy [1,11]. Experimental studies have described histological evidence of pulmonary hemorrhage after the injection of viperid venoms [12] and purified toxins, especially metalloproteinases [13,14], but also other venom components [15,16]. Viperid venoms have also been reported to affect pulmonary mechanical parameters [12,17]. However, few studies have shown pulmonary effects induced by venoms or toxins devoid of hemorrhagic activity [18,19,20,21].

The venoms of the predominantly Australian elapid genus *Pseudechis* (black snakes) are rich in phospholipases A_2_ (PLA_2_s) but have a low content of three-finger toxins (3FTxs) [22], and proteomic analysis of the venom of the Papuan black snake, *Pseudechis papuanus*, a species endemic to Papua New Guinea, Indonesian Papua, and Australia’s Torres Strait Islands, revealed a high abundance (~90%) of PLA_2_s and a low content of 3FTxs (3.1%) and class P-III metalloproteinases (2.8%) [21,23]. PLA_2_s are hydrolytic enzymes that cleave the *sn*-2 ester bond of glycerophospholipids and play key roles in the overall toxicity of snake venoms [24]. *P. papuanus* venom induced severe acute pulmonary alterations after intravenous injection in mice. These changes were associated with widespread edema in the alveolar spaces [21], and this effect was abrogated when PLA_2_ activity was inhibited with *p*-bromophenacyl bromide [21]. Thus, the investigation of the pulmonary action of this PLA_2_-rich venom constitutes a model for assessing the mechanisms of PLA_2_-induced pulmonary damage, which in turn may shed light on various pathophysiological alterations occurring in other pulmonary diseases.

Group IA PLA_2_ from the venom of the cobra *Naja sputatrix* induced pulmonary edema after i.v. injection and intratracheal instillation in rats [19]. Endogenously secreted PLA_2_s are upregulated in acute respiratory distress syndrome (ARDS), a severe clinical condition that has a variety of etiologies [25], and in preclinical models of lung injury [26,27]. Moreover, the concentrations of group IIA inflammatory PLA_2_ are increased in bronchoalveolar lavage fluid (BALF) in ARDS [28] and secretory PLA_2_s have been proposed as biomarkers of ARDS [29]. The main aims of this work were to study the pulmonary pathological and pathophysiological effects induced by the PLA_2_-rich venom of *P. papuanus,* and to assess the role of venom PLA_2_s in these effects. Our observations offer insights on the possible mechanisms by which PLA_2_s cause pulmonary damage.

## 2. Results

### 2.1. Macroscopic and Histological Alterations in the Lungs and Inhibition by Varespladib

**Figure 1:**
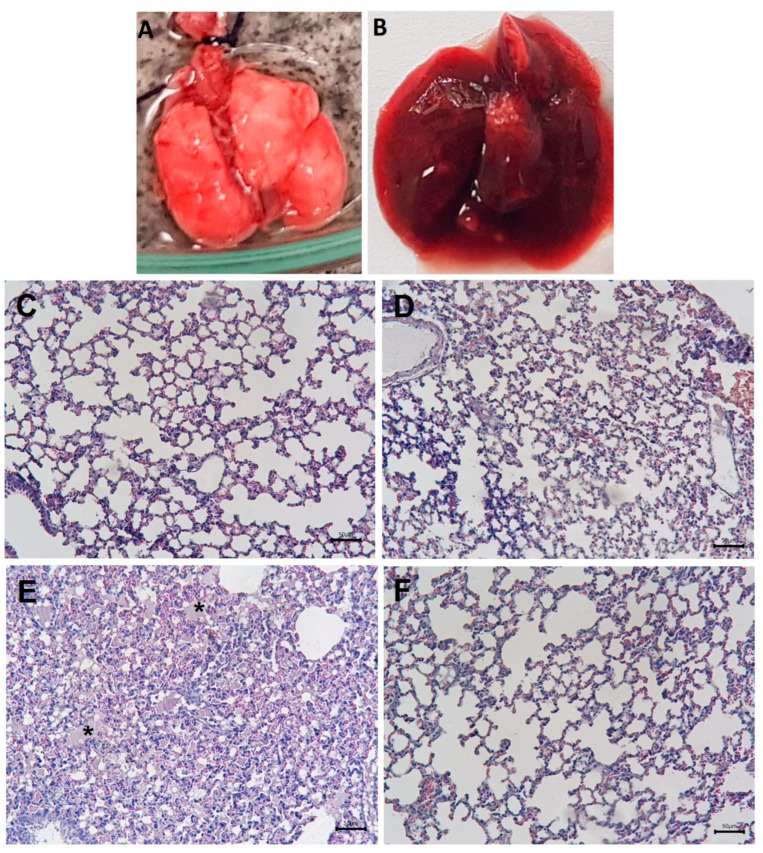
Macroscopic and microscopic alterations induced by *P. papuanus* venom in mouse lungs. (**A**) Lungs from mice receiving saline solution (SS). (**B**) Lungs from mice injected with 35 µg of venom. (**C**–**F**) Light micrographs of pulmonary tissue from mice injected with SS (**C**), 15 µg of venom (**D**), 35 µg of venom (**E**), and 35 µg of venom preincubated with varespladib (500 µM) (**F**). Venom (dissolved in 100 µL of SS) was injected i.v. via the caudal vein. Control mice received 100 µL of SS. After 40 or 60 min, the mice were euthanized with isoflurane, the thoracic cavity was opened, and the lungs were excised. The lungs were then sectioned, fixed in formalin, and processed for embedding in paraffin. Sections (4 µm thick) were cut and stained with hematoxylin-eosin. A normal histological appearance was observed in samples from mice receiving SS or 15 µg of venom, whereas abundant hyaline material (*) was present in the alveolar spaces of mice receiving 35 µg of venom. This effect was completely abrogated when venom was preincubated with varespladib. Scale bars: 50 µm.

Control mice that received an i.v. injection of 0.15 M NaCl (saline solution, SS) displayed pulmonary tissue with a normal macroscopic and histological appearance (Figure 1A,C). Mice injected with 15 µg of venom did not develop major pulmonary histological alterations by 1 h after injection (Figure 1D), whereas those injected with 35 µg of venom were sacrificed at 40 min because previous observations showed that, with this dose, mice died after approximately 40 to 60 min. Macroscopic examination of these mice revealed prominent congestion and a reddish appearance of the lungs (Figure 1B). Histologically, abundant hyaline material was observed in the alveolar spaces, indicating pulmonary edema (Figure 1E). These macroscopic and histological alterations were completely abolished when venom was incubated with varespladib before injection (Figure 1F). The mice treated with varespladib survived the 1 h observation period, without showing signs of toxicity. Likewise, varespladib inhibited the PLA_2_ activity of venom towards the substrate NOBA. The incubation of the venom with varespladib concentrations of 125, 250 and 500 µM inhibited the PLA_2_ activity by 81 ± 18%, 93 ± 26% and 95 ± 19% (n = 3), respectively, compared to the enzyme activity of the venom without the inhibitor. Based on these results, a varespladib concentration of 500 µM was used throughout the rest of the study to assess the role of PLA_2_ in the venom-induced effects.

### 2.2. Effect of Venom on Lung Surfactant Factor

**Figure 2:**
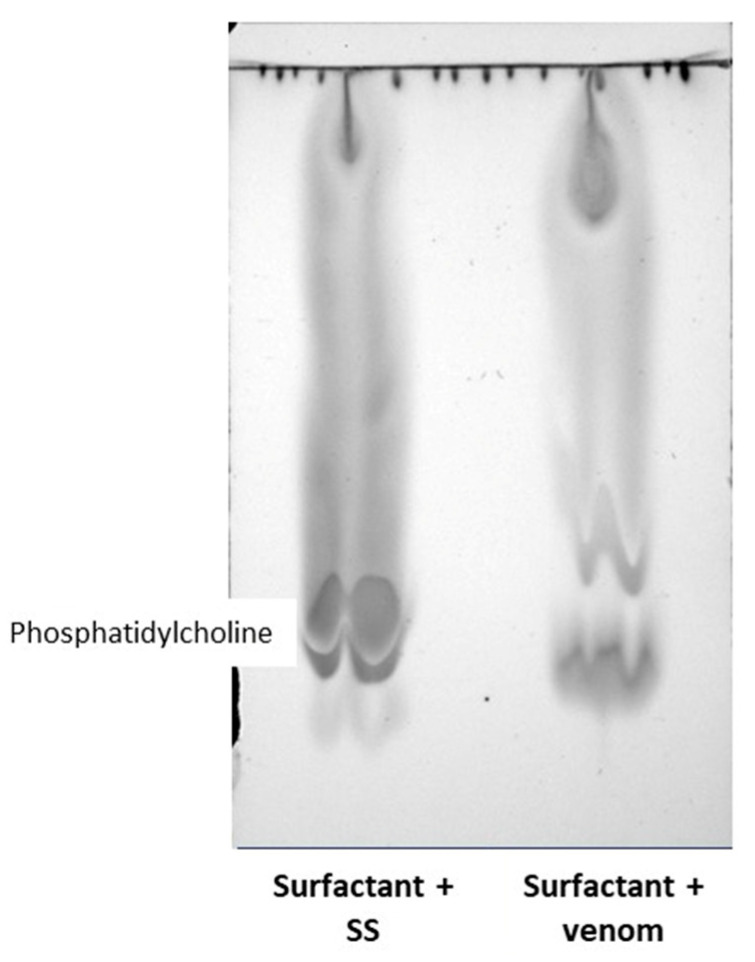
Degradation by *P. papuanus* venom of phosphatidylcholine in bovine lung-derived surfactant factor. Surfactant factor was incubated with either venom or SS. Phospholipids were then extracted, separated by thin-layer chromatography (TLC), and visualized (see Section 4.5 for details). Left: surfactant factor incubated with SS; right: surfactant factor incubated with venom. The label phosphatidylcholine indicates the position where this phospholipid migrates.

TLC analysis of lung surfactant factor incubated with SS revealed a major spot corresponding to phosphatidylcholine, the main component of this surfactant material. When the surfactant was incubated with 10 µg of venom for 30 min at 37 °C and the sample then analyzed by TLC, this main spot was absent, indicating the degradation of this component by the venom (Figure 2).

### 2.3. Cytotoxic Effect of Venom on Endothelial Cells

**Figure 3:**
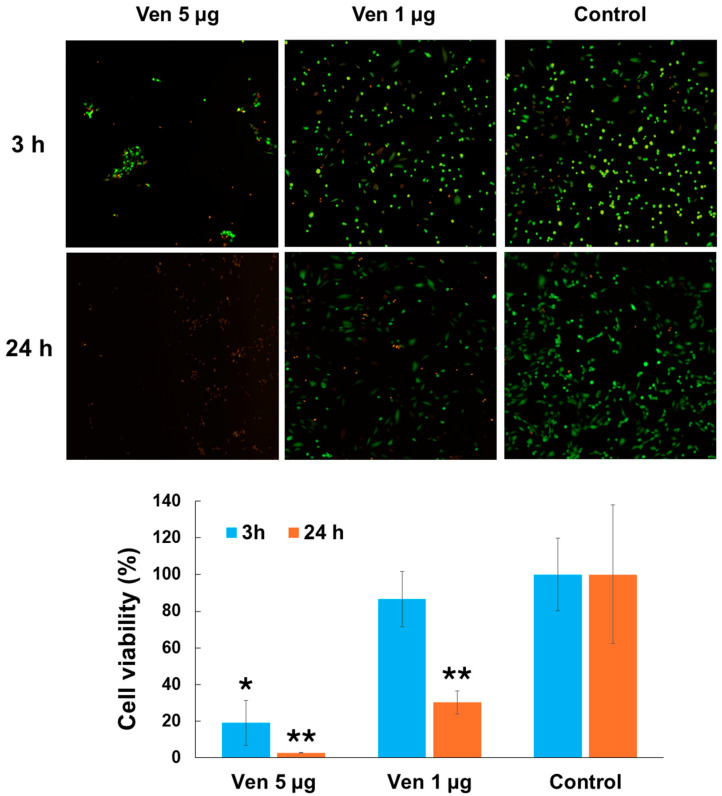
Cytotoxicity of *P. papuanus* venom on endothelial cells in vitro. HUVEC monolayers grown on 96-well plates were incubated with 5 µg or 1 µg of venom for 3 h and 24 h at 37 °C. Wells containing cells with ECGM (endothelial cell growth medium) alonewere used as controls (100% viability). Viability was assessed with a LIVE/DEAD viability/cytotoxicity assay kit for mammalian cells (see Section 4.6 for details). Fluorescence at 530 nm was recorded as an index of cell viability, and images were captured using a Cytation 5 Cell Imaging Multimode Reader. Cell viability is shown as the mean ± SD. Experiments were performed in triplicate. * *p* < 0.001 when compared with 3 h control, ** *p* < 0.001 when compared with 24 h control.

*P. papuanus* venom was cytotoxic to cultured HUVEC, as assessed by the decrease in absorbance at 530 nm and the detachment of cells from their substrate (Figure 3). Cytotoxicity was observed with 1 µg of venom only after 24 h, whereas with 5 µg of venom cytotoxicity was seen after 3 h and 24 h.

### 2.4. Effect of Venom on Pulmonary Mechanics

**Figure 4:**
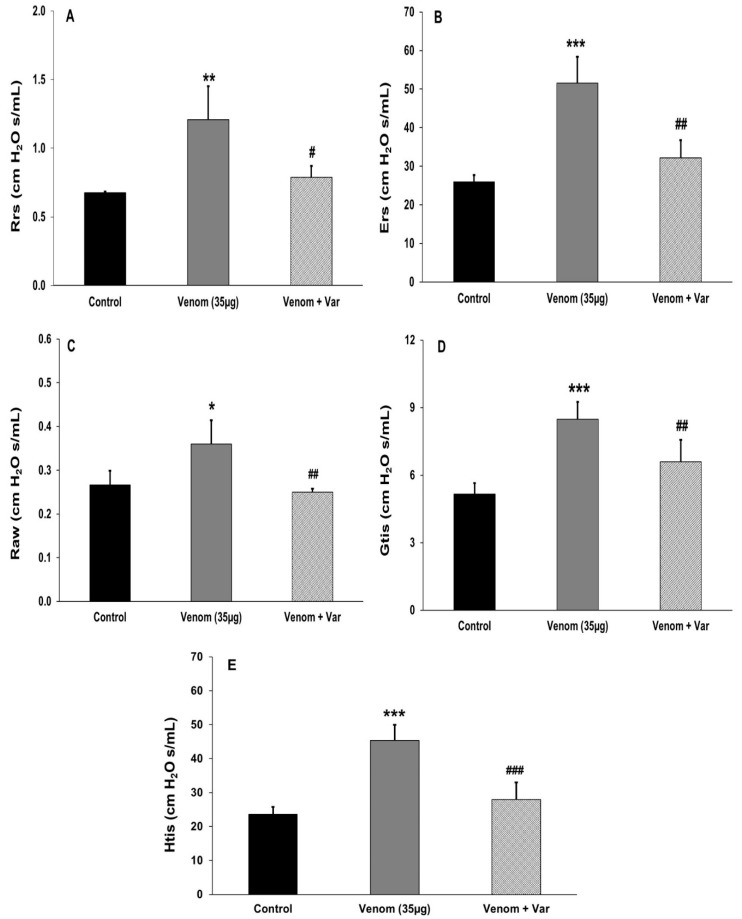
Alterations in the pulmonary mechanical parameters of mice injected with *P. papuanus* venom and their inhibition by varespladib. Mice received an i.v. injection of SS (control), venom (35 µg), or venom (35 µg) preincubated with varespladib (Var, 500 µM) (see Section 4.7 for details). Thirty minutes after injection, the mice were anesthetized with pentobarbital sodium (50 mg/kg, i.p.), and a plastic cannula (caliber 20) was introduced into the trachea. The mice were then ventilated with a volume of 10 mL/kg at a respiratory frequency of 120 cycles/min and using a sinusoidal curve of inspiratory flux. The mechanical parameters evaluated were the resistance (Rrs) (**A**) and elastance (Ers) (**B**) of the respiratory system, the resistance of the airways (Raw) (**C**), and the resistance (Gtis) (**D**) and elastance (Htis) (**E**) of the tissue. The columns represent the mean ± SD (n = 3 for controls and 4–6 for venom and venom + varespladib). * *p* < 0.05, ** *p* < 0.01 and *** *p* < 0.001 compared to control mice injected with SS. ^#^ *p* < 0.05, ^##^ *p* < 0.01 and ^###^ *p* < 0.001 compared to venom alone.

*P. papuanus* venom (35 µg, i.v.) increased all of the pulmonary parameters examined 30 min after injection compared to mice injected with SS (Figure 4). Preincubation of the venom with varespladib abrogated these mechanical alterations and their values approached those of SS-treated (control) mice (Figure 4). Thus, venom exerted a marked effect on pulmonary mechanical parameters in mice.

### 2.5. Effect of Venom on Tracheal and Bronchial Reactivity

**Figure 5:**
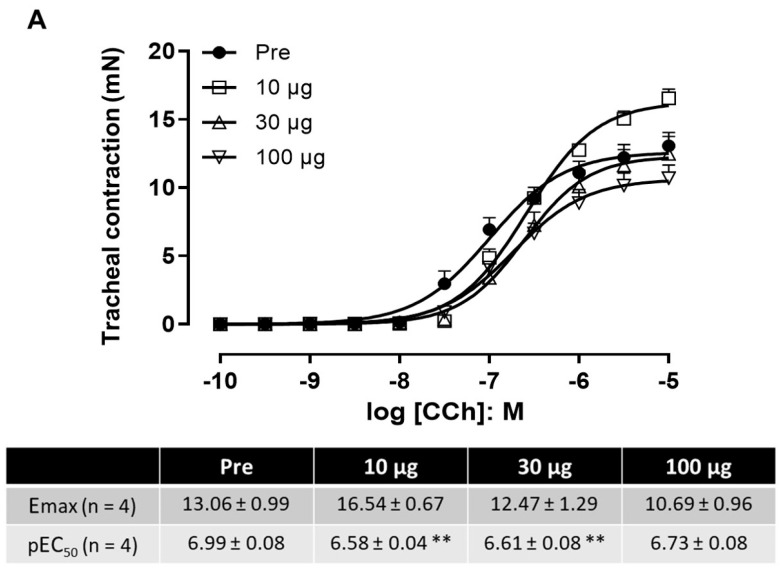

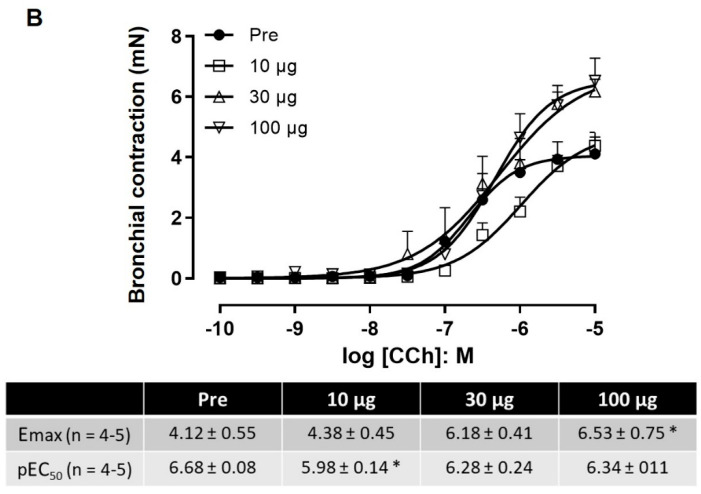
Influence of *P. papuanus* venom on contractile responses of mouse tracheal (**A**) and bronchial (**B**) rings to muscarinic agonist carbachol. Parameters evaluated included maximal contraction obtained (E_max_) and concentration of agonist that produced 50% of maximal response (EC_50_). Preincubation of tissues with different concentrations of venom (10, 30 and 100 µg/mL) had no consistent effect on two parameters examined. Points represent mean ± SD (n = 4). * *p* < 0.05 and ** *p* < 0.01 compared to control curves (Pre) obtained before incubation with venom. See Section 4.8 for full experimental details.

The maximal contraction (E_max_) and the concentration of agonist (carbachol) that induced 50% of the maximal response (EC_50_) were determined in tracheal and bronchial rings after 10 min incubation with different venom concentrations (Figure 5). In tracheal tissue, there were no significant differences in the E_max_ values, despite the increase seen with the lowest venom concentration (10 µg/mL) compared to the control group (Figure 5A). Slight but significant rightward shifts (0.38–0.41 log_10_ units) in EC_50_ were seen in the concentration–response curves to carbachol at venom concentrations of 10 and 30 µg/mL, but not with the highest concentration (100 µ/mL). In bronchial tissue, there was a 50–58% increase in the E_max_ with the two highest venom concentrations. This was significant at the highest venom concentration (100 µg/mL). Conversely, the EC_50_ values were generally unchanged, except for a rightward shift of 0.7 log_10_ units with the lowest venom concentration (Figure 5B). Overall, the changes observed in the E_max_ and EC_50_ values were not consistent among venom concentrations or between tissues, implying that the venom did not have a major effect on tracheal and bronchial contractility.

### 2.6. Effect of Venom on Exhaled NO

There were no significant differences (*p* > 0.05) in the concentrations of exhaled NO between SS-treated (control) mice (23 ± 10 ppb) and those receiving 35 µg of venom (14 ± 3 ppb) or 35 µg of venom preincubated with 500 µM varespladib (15 ± 6 ppb) (n = 4–6).

### 2.7. Effect of Venom on Angiotensin-Converting Enzyme Activity (ACE)

**Figure 6:**
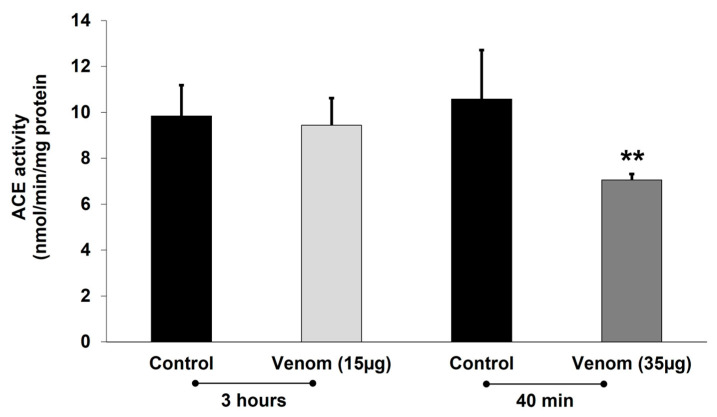
Effect of *P. papuanus* venom on angiotensin-converting enzyme (ACE) activity of mouse pulmonary tissue. Mice received i.v. injection of either saline solution (SS, control) or venom (15 µg or 35 µg) dissolved in SS and were euthanized either 40 min or 3 h later, after which lungs were removed and homogenized, and ACE activity was determined (see Section 4.10 for full experimental details). Columns represent the mean ± SD (n = 5 for controls and 7–8 for venom-treated mice). ** *p* < 0.01 compared to SS-injected (control) mice.

ACE activity was quantified in homogenates taken from the lungs of mice euthanized 40 min or 3 h after the i.v. injection of SS or venom (15 µg or 35 µg). The lowest dose of venom (15 µg) had no effect on pulmonary ACE activity, whereas a significant decrease in this activity was seen in mice injected with 35 µg of venom (Figure 6).

### 2.8. Analyses of BALF

#### 2.8.1. Inflammatory Cells

There were no significant differences in the total cell counts in BALF in mice treated with 35 µg of venom alone for 40 min compared to SS-treated mice or mice treated with 35 µg of venom preincubated with varespladib (Supplementary Figure S1). These results indicate that the venom did not increase the number of inflammatory cells in BALF.

#### 2.8.2. Total Protein and Hemoglobin Concentration

**Figure 7:**
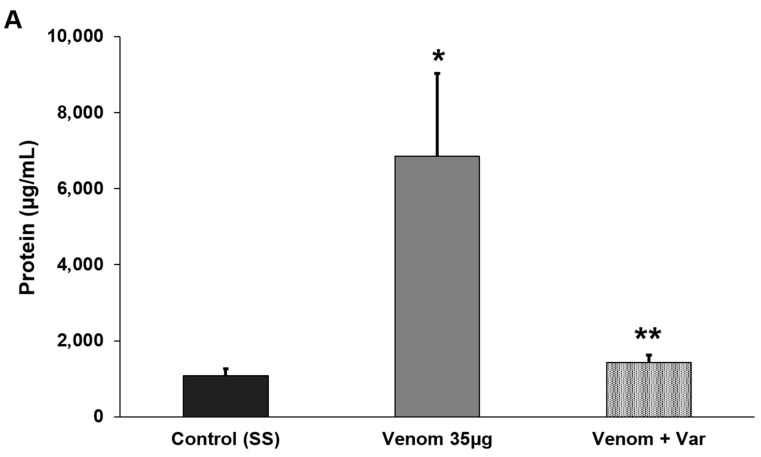

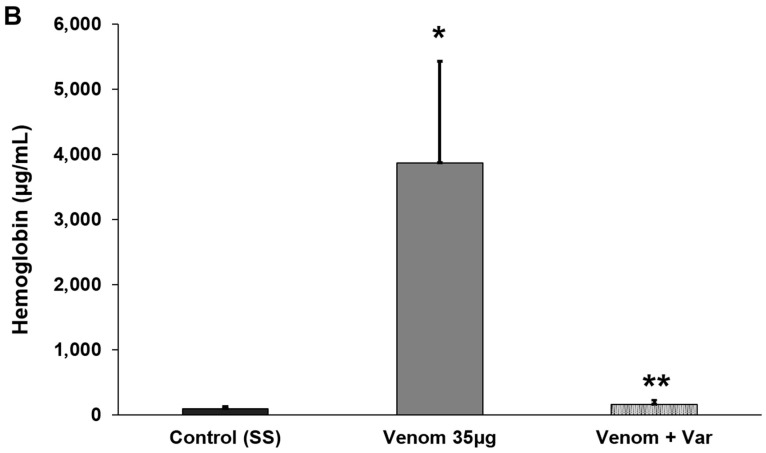
Total protein (**A**) and hemoglobin (**B**) concentrations in bronchoalveolar lavage fluid (BALF) collected from mice injected with either saline solution (SS, control), venom (35 µg) or venom (35 µg) preincubated with 500 µM varespladib. The mice were injected with SS or venom and, after 40 min, were euthanized and exsanguinated, and the trachea was exposed and cannulated. Subsequently, 300 µL of cold SS was flushed through the cannula and the liquid was collected. This process was repeated five times and approximately 1.5 mL of BALF was collected. After centrifugation, the supernatant was collected and the total protein and hemoglobin concentrations were determined (see Section 4.11 for full details). The columns represent the mean ± SD (n = 15 for venom and n = 5 for control and venom + varespladib). * *p* < 0.05 compared to SS-treated (control) mice. ** *p* < 0.05 compared to venom alone.

There was a significant increase in the concentration of total proteins and hemoglobin in BALF collected 40 min after i.v. injection of 35 µg of venom when compared to BALF from SS-treated (control) mice. The preincubation of the venom with varespladib completely abrogated this effect since the total protein and hemoglobin concentrations in BALF were not different from those in SS-treated mice (Figure 7). These findings show that the venom caused the extravasation of a protein-rich fluid through the alveolar–capillary barrier into the alveolar spaces.

### 2.9. Effects of Anti-Inflammatory Agents on Venom-Induced Pulmonary Edema

**Figure 8:**
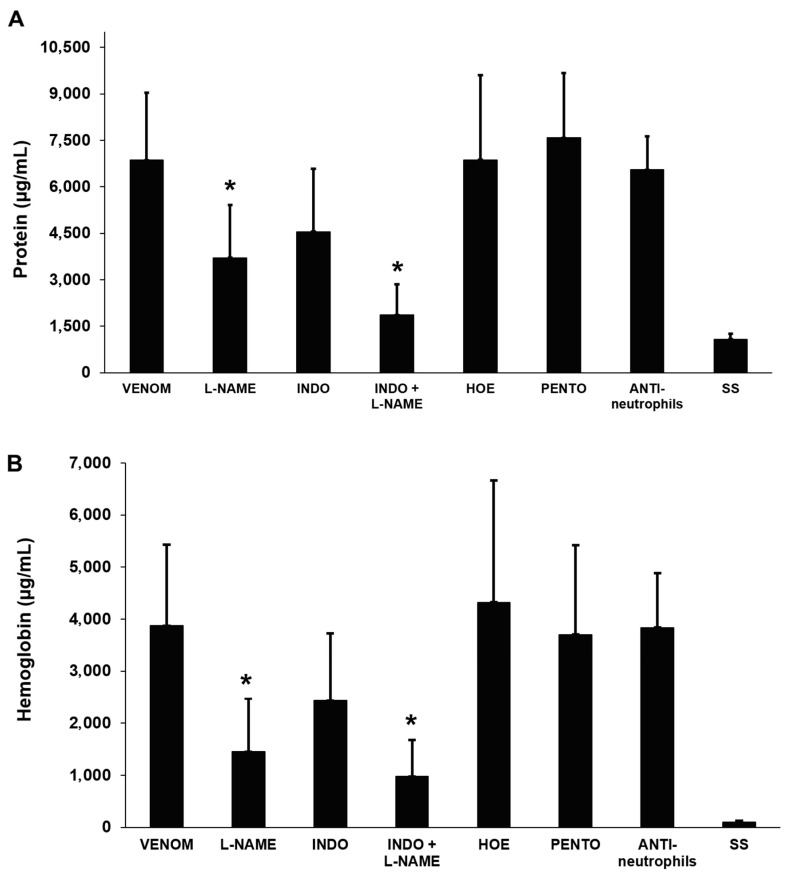
Effects of anti-inflammatory agents on the total protein (**A**) and hemoglobin (**B**) concentrations in bronchoalveolar lavage fluid (BALF) from mice injected with saline solution (SS, control) or venom (35 µg). Negative controls were injected with SS alone and positive controls received venom alone. For the remaining groups, mice were pretreated i.p. with L-NAME, indomethacin (INDO), HOE-140 (HOE), pentoxifylline (PENTO), or anti-neutrophil antibodies (Section 4.12 for details of doses and times of administration). The mice were euthanized and exsanguinated 40 min after venom injection, and the trachea was exposed and cannulated. Subsequently, 300 µL of cold SS was flushed through the cannula and the liquid was collected. This process was repeated five times and approximately 1.5 mL of BALF was recovered. After centrifugation, the total protein and hemoglobin concentrations of the supernatants were determined. The columns represent the mean ± SD (n = 15 for venom and 4–8 for saline solution (SS) and pretreatments with inhibitors). * *p* < 0.05 compared to mice injected with venom alone.

Of the various anti-inflammatory agents tested against venom (35 µg)-induced pulmonary edema, only the NO synthase inhibitor L-NAME significantly attenuated the venom-induced increase in the total protein and hemoglobin concentrations of BALF 40 min after envenoming (Figure 8). There was a tendency for indomethacin, a COX inhibitor, to normalize these parameters, but the effect was not significant. Similarly, a combination of L-NAME and indomethacin also tended to improve the normalization of these parameters, but the responses were not significantly different from those seen with L-NAME alone. This finding indicates that NO plays a role in the venom-induced fluid accumulation in alveolar spaces. The raw data used to prepare the graphs of this publication are included in Supplementary Table S1.

## 3. Discussion

The venom of *P. papuanus* induces a complex pattern of pathological and pathophysiological alterations in the lungs of mice characterized by the formation of widespread edema in the alveolar spaces, with the accumulation of a protein-rich exudate that may affect gas exchange in the lungs. High amounts of hemoglobin were present in BALF as a consequence of venom-induced intravascular hemolysis and an increase in the permeability of the capillary–alveolar barrier. In addition, the venom hydrolyzed phospholipids in a surfactant factor preparation, was cytotoxic to endothelial cells, and affected the mechanical properties of the respiratory system. Alterations in the mechanical properties included an increase in the resistance and elastance of the respiratory system and distal lung tissue, thereby reducing compliance, but without markedly affecting the tracheal and bronchial contractility. The lack of a consistent effect on the contractility of the latter tissues suggests that the venom PLA_2_ caused minimal damage to the intactness of the contractile mechanisms of the muscles involved. These findings suggest that the action of the venom is likely to be centered at the capillary–alveolar interface and associated with an increase in the permeability of this barrier, thereby leading to pulmonary edema. Our observations bear resemblances to some features of animal models of ARDS, including an increase in the permeability of the capillary–alveolar barrier and alterations in pulmonary mechanical parameters. However, a key feature of ARDS, i.e., the presence of an inflammatory infiltrate in the lungs [30,31], was not observed in our model. This finding likely reflects the rapid onset of alterations that occur within 40 min, with mice dying before the accumulation of leucocytes in the lung parenchyma.

Other venoms and isolated venom components have been described as inducing pulmonary alterations both clinically and experimentally. Hemorrhagic viperid venoms and toxins, i.e., metalloproteinases and PLA_2_s, induce pulmonary hemorrhage and edema, associated with acute lung injury [13,14,32,33,34] with alterations in mechanical respiratory parameters [12]. Severe scorpion envenoming is frequently associated with pulmonary edema that occurs secondarily to myocardial injury and cardiogenic shock as a consequence of the massive release of catecholamines [35,36], although it is likely that other mechanisms are also involved [37]. ARDS-like manifestations also occur in envenoming via mass bee attacks [38,39]. Thus, a variety of venoms induce lung injury, as recently reviewed [40], but the mechanisms involved are likely to vary depending on the venom.

Since *P. papuanus* venom is highly rich in PLA_2_s [21,22,23], we examined the role of PLA_2_ in the venom-induced pulmonary alterations by using varespladib, an inhibitor that has been shown to abrogate PLA_2_ activity in a variety of snake venoms [41,42]. Varespladib completely abrogated the effects of *P. papuanus* venom in the lungs. These observations agree with a previous study in which PLA_2_ activity was inhibited by chemical modification with *p*-bromophenacyl bromide [21]. The role of venom PLA_2_ in the pathogenesis of pulmonary damage is compatible with observations showing that endogenously secreted group IIA PLA_2_ is upregulated in experimental models of ARDS and that the concentration of this enzyme is elevated in BALF in ARDS [26,27,28]. In addition to PLA_2_s, which comprise 90% of *P. papuanus* venom [21], this venom contains low amounts of metalloproteinases, cysteine-rich secretory proteins (CRISPs), three-finger neurotoxins, and L-amino acid oxidases [21]. However, it is very unlikely that these proteins contributed to the pulmonary effects described here since the PLA_2_ inhibitor varespladib completely abrogated the venom-induced alterations, a finding that highlights the central role of PLA_2_ in this model.

*P. papuanus* venom PLA_2_s might contribute to pulmonary alterations through several mechanisms. One mechanism may involve the direct cytotoxic action of PLA_2_s on endothelial cells and pneumocytes. Indeed, as shown here, the venom was cytotoxic to endothelial cells (HUVECs) in culture and this cytotoxicity could directly affect the integrity of the capillary–alveolar barrier, thus contributing to the alveolar accumulation of fluid. Although a few studies have examined the cytotoxicity of snake venoms and toxins on cultured pulmonary endothelial cells (e.g., [43,44]), we did not have access to pulmonary endothelial cells during this study. Hence, HUVECs were chosen as surrogate cells to examine the endothelial cell cytotoxicity of *P. papuanus* venom since this cell line has been widely used to study the effects of snake venoms and toxins in general [45,46,47,48,49,50,51], including venom PLA_2_ [52,53,54,55,56,57]. While we are aware that the use of pulmonary endothelial cells would have been more appropriate, we believe that our results provide evidence that *P. papuanus* venom would also be cytotoxic to pulmonary endothelial cells.

A second mechanism of *P. papuanus* venom-induced pulmonary damage could involve the degradation of surfactant factors since, as shown here, the venom degraded phospholipids in the preparation of surfactant factors. Venom PLA_2_ are likely to reach the alveolar space and hydrolyze surfactant factors following an increase in vascular permeability or damage to endothelial cells and pneumocytes. The surfactant factor, which covers the alveolar surface and reduces surface tension [58], is composed of lipids and proteins, with a high concentration of phosphatidylcholine [59]. The hydrolysis of these phospholipids would increase surface tension, thereby causing a decrease in lung distensibility that could in turn contribute to pathophysiology.

A third possible mechanism of action of PLA_2_ in this model relates to the release of arachidonic acid from cellular phospholipids, with the consequent synthesis of prostaglandins. Although prostaglandins have been used to treat pulmonary conditions such as ARDS because of their vasodilatory action, which increases oxygenation [60], in the case of *P. papuanus* venom, such vasodilation could potentiate the edema caused by venom PLA_2_. However, the pretreatment of mice with indomethacin, a COX inhibitor, did not significantly affect the total protein and hemoglobin concentrations in BALF. This finding suggests that either prostaglandins play a minor role in this model or that the rapid onset of the alterations seen here preceded and surpassed the action of prostaglandins.

PLA_2_ may also contribute to the venom-induced pulmonary damage in an indirect way. The toxicologic profile of this venom goes beyond the pulmonary alterations described here and in a previous report [21] as this venom also induces myotoxicity, inhibits platelet aggregation, causes intravascular hemolysis and has an anticoagulant effect, all of which depend on the action of PLA_2_s [21,23,61]; this venom is also neurotoxic experimentally and clinically [62,63,64]. The intravascular hemolysis described in mice [21], with the accumulation of large amounts of hemoglobin in plasma and urine and extravasated fluid in the alveoli, is likely to have a direct deleterious effect in the lungs. Free hemoglobin contributes to lung injury [65] through several mechanisms that include NO scavenging, endothelial activation, inflammation, and oxidative injury [66,67]. Thus, in addition to the direct cytotoxic effect on endothelial cells, and possibly on pneumocytes, venom PLA_2_s may also exert an indirect deleterious effect through their hemolytic effect and the action of released hemoglobin in the lungs.

The role of several inflammatory mediators in the accumulation of proteinaceous fluid in BALF was assessed using specific inhibitors. However, no significant beneficial effect was observed when mice were pretreated with inhibitors of COX, TNF-α, bradykinin, and neutrophils. Thus, although these inflammatory mediators, pathways, and cells have been shown to play a role in ARDS [68,69,70,71], the lack of effect of the inhibitors tested here could be explained by the rapid onset of pulmonary responses after the injection of *P. papuanus* venom; these alterations may well occur before the synthesis of these mediators and the recruitment of neutrophils. The only drug that reduced the concentration of protein in BALF was L-NAME, suggesting that NO is involved in the pathogenesis of this effect. In this regard, the lack of increase in exhaled NO in our model might be related to the accumulation of edema fluid in the alveolar spaces that could affect the diffusion of locally synthesized NO to the exhaled air. Similar observations have been made in patients with ARDS [72].

NO may contribute to pulmonary effects in this model, either through its direct vasodilatory effects, or by generating highly reactive molecules, i.e., peroxynitrites [73], that may damage endothelial cells or pneumocytes. NO is also known to affect the expression of surfactant protein-B [74]. Our observations are in line with previous studies showing that NO plays a role in experimental models of lung injury [74,75,76]. In our model, the observed deleterious effect of NO may depend on the fact that vasodilation, in the context of damaged microvasculature, may result in increased plasma extravasation to the alveolar space. *P. papuanus* venom also reduced the activity of ACE in lung homogenates at the highest amount tested (35 µg). ACE generates angiotensin II, a vasoconstrictor agent, and hydrolyzes bradykinin, a potent vasodilator [77]. Thus, the reduction in ECA activity observed here may contribute to vasodilation that in turn may play a role in the venom-induced increase in vascular permeability and edema. However, the lack of effect of HOE, a bradykinin B2 receptor antagonist, on the total protein and hemoglobin concentrations of BALF suggests that this peptide is not involved in the alterations in these parameters.

In conclusion, we developed a murine experimental model of acute pulmonary pathology based on the i.v. injection of *P. papuanus* venom. This acute severe response reflects the action of venom PLA_2_ through several possible non-mutually exclusive mechanisms that include (a) the direct cytotoxic action of PLA_2_ on endothelial cells and possibly pneumocytes; (b) the enzymatic hydrolysis of pulmonary surfactant factor phospholipids; (c) the deleterious action of hemoglobin in the lungs as a consequence of venom-induced intravascular hemolysis; and (d) the action of NO synthesized as part of the inflammation elicited by the venom. The relative role of each of these mechanisms in the observed effect demands further investigation. From a more general perspective, this model could be useful for understanding the mechanisms by which PLA_2_s affect the capillary–alveolar barrier and induce the accumulation of proteinaceous fluid in alveoli in ARDS and other pathophysiological pulmonary conditions.

## 4. Materials and Methods

### 4.1. Venom

The venom of adult specimens of *P. papuanus* was kindly provided by Dr. David Williams. Upon collection, the venom was freeze-dried and maintained at −20 °C until use. Venom solutions were prepared in sterile 0.15 M NaCl (saline solution, SS) before each experiment.

### 4.2. Animal Ethics Approvals

Experiments were performed in Costa Rica using CD-1 mice (18–20 g) of both sexes, provided by the Bioterium of Instituto Clodomiro Picado. During the experiments, the mice were housed at 22–24 °C and 60–65% humidity, on a 12 h light/dark cycle, with ad libitum access to food and water. The experimental protocols involving mice in Costa Rica were approved by the Institutional Committee for the Care and Use of Laboratory Animals (CICUA) of the University of Costa Rica (approval number CICUA-035-2021). For the experiments performed in Brazil, BALB/c mice (20–21 g) of both sexes were provided by the Central Animal House (Centro Multidisciplinar para Investigação Biológica—CEMIB) of the State University of Campinas (UNICAMP). The experimental protocols were approved by the institutional Committee for Ethics in Animal Use (CEUA-UNICAMP, approval numbers 4638-1/2017 and 4668-1/2017) and were conducted according to the general ethical guidelines for animal use established by the Brazilian Society of Laboratory Animal Science (SBCAL) and Brazilian legislation (Federal Law no. 11794, of 8 October 2008), in conjunction with the guidelines for animal experiments established by the Brazilian National Council for the Control of Animal Experimentation (CONCEA) and EU Directive 2010/63/EU for the Protection of Animals Used for Scientific Purposes.

### 4.3. Histological Analyses

Groups of CD-1 mice (18–20 g) received intravenous (i.v., caudal vein) injection of 15 µg or 35 µg of venom dissolved in 100 µL of SS. Control mice received the same volume of SS. One hour after injection (in the case of mice injected with SS or 15 µg venom) or 40 min after injection (in the case of mice receiving 35 µg venom because at this dose mice died after this time interval), the mice were euthanized with inhaled isoflurane. After death, the thoracic cavity was opened and the lungs were removed, washed briefly with SS, sectioned into smaller portions, and immediately placed in formalin fixative solution. After routine processing, the tissues were embedded in paraffin and sections 4 µm thick were prepared and stained with hematoxylin-eosin for histological examination. To assess the role of PLA_2_ activity in the venom-induced pulmonary histological alterations, venom preincubated with the PLA_2_ inhibitor varespladib (500 µM, see next section) was injected i.v. into mice (venom amount: 35 µg), as described above. One hour after injection, the mice were sacrificed and samples of pulmonary tissue were collected and processed as described.

### 4.4. Phospholipase A_2_ (PLA_2_) Activity and Inhibition by Varespladib

The PLA_2_ activity of the venom was assayed using the method described by [78] after adaptation for microplates. We used 4-nitro-3 (octanoyloxy) benzoic acid (NOBA) as substrate. Absorbances were recorded at 425 nm in a microplate reader. For the inhibition of PLA_2_ activity, a solution of venom (30 µg) in SS was incubated with either SS or various concentrations (125 µM, 250 µM and 500 µM) of varepladib [LY315920, provided by Ophirex, Inc. (Corte Madera, CA, USA) and prepared by ChemiTek (Indianapolis, IN, USA)] for 30 min at 37 °C and the residual enzymatic activity was then assessed as described above.

### 4.5. Effect of Venom on Lung-Derived Surfactant Factor

The ability of *P. papuanus* venom to hydrolyze the phospholipids of surfactant factors was assessed using a bovine lung-derived surfactant factor (Survanta^®^, Abbvie, Inc., Chicago, IL, USA) that consisted of phosphaditylcholine (25 mg/mL), triglycerides (0.5–1.75 mg/mL), free fatty acids (14 mg/mL), and proteins (0.1–1 mg/mL). Venom (10 µg in 10 µL of SS) was added to 100 µL of a suspension containing 2.5 mg of surfactant factor. A control tube included the same amount of surfactant incubated with 10 µL of SS. After 30 min of incubation at 37 °C, 1 mL of a methanol–chloroform mixture (2:1 *v*/*v*) was added. This was followed by the addition of 1 mL of chloroform and 1 mL of distilled water, and the sample was vortexed. After centrifugation (2500× *g*, 5 min), the chloroform layer was collected and the samples were dried in a vacuum centrifuge. The samples were subsequently resuspended in 50 µL of chloroform and separated by thin-layer chromatography (TLC) on precoated silica gel plates using a chloroform–methanol–acetic acid–water mixture (54:25:8:4, *v*/*v*) as solvent. The lipids on the TLC were visualized in a chamber containing iodine.

### 4.6. Cytotoxic Effect of Venom on Endothelial Cells

Human umbilical vein endothelial cells (HUVECs, Cell Applications Inc., San Diego, CA, USA), passaged 4 to 5 times, were cultured in a T25 culture flask with endothelial cell growth medium (ECGM, Cell Applications, Inc.) at 37 °C and 5% CO_2_ until reaching approximately 80% confluence. HUVEC monolayers were trypsinized, resuspended in ECGM, and plated on 96-well plates at a density of 5000 cells/well. Cells were incubated for 24 h at 37 °C and 5% CO_2_; then, solutions containing 5 µg or 1 µg of *P. papuanus* venom in 100 µL of ECGM were applied to each well in triplicate. Wells containing ECGM only were used as 100% viability controls, and wells containing 0.1% Triton-X-100 in ECGM were used as 0% viability controls. The plate was incubated for either 3 h or 24 h at 37 °C, and cell viability and cytotoxicity were evaluated using a LIVE/DEAD viability/cytotoxicity assay kit for mammalian cells (ThermoFisher Scientific, Waltham, MA, USA) based on the manufacturer’s protocol. Briefly, the medium was removed, the wells were washed three times with SS, and the staining solution containing 1 µL of 2 µM calcein AM and 5 µL of 4 µM ethidium homodimer-1 in 2 mL SS was added to each well and incubated for 30 min at room temperature. Viable cells with intact membranes presented the green fluorescence of the cytoplasm, whereas cells with disruptions to their membrane showed orange staining of the nuclei. Fluorescence was measured at 645 nm and 530 nm, and images were captured using a Cytation 5 Cell Imaging Multimode Reader (Agilent, Santa Clara, CA, USA).

### 4.7. Effects of Venom on Pulmonary Mechanics

Three groups of BALB/c mice (20–21 g) were used: Group 1 (control) received an i.v. injection of 100 µL of SS, Group 2 received an i.v. injection of 35 µg venom dissolved in 100 µL of SS, and Group 3 received an i.v. injection of 35 µg venom that had been preincubated with 500 µM varespladib in a total volume of 100 µL, as described above. Thirty minutes after injecting venom, the mice were anesthetized (sodium thiopental, 70 mg/kg, i.p.), paralyzed (pancuronium, 0.2 mg/kg, i.v.), and tracheostomized. The mice were then connected to a mechanical ventilator for small animals (FlexiVent, Scireq, Montreal, QC, Canada) and were ventilated with a volume of 10 mL/kg, at a respiratory frequency of 120 cycles per min, and using a sinusoidal curve of inspiratory flux. The following mechanical parameters were evaluated: the resistance (Rrs) and elastance (Ers) of the respiratory system, resistance (Gtis) and elastance (Htis) of the tissue, and resistance of aereal ways (Raw). The parameters of oscillatory mechanics were determined as described elsewhere [79,80].

### 4.8. Effect of Venom on Tracheal and Bronchial Reactivity

Groups of BABL/c mice (20–21 g) were euthanized by CO_2_ inhalation and exsanguinated by sectioning the abdominal thoracic artery. The tracheae and bronchi were removed and placed in a Krebs–Henseleit solution (composition, in mM: 118 NaCl, 4.7 KCl, 2.4 CaCl_2_, 1.2 MgSO_4_, 1.2 KH_2_PO_4_, 25 NaHCO_3_ and 11 glucose). After removing the connective tissue, 2 mm rings were cut and mounted in a myograph (AD Instruments, NSW, Australia) containing 5 mL of Krebs–Henseleit solution with the continuous bubbling of a carbogen mixture (95% O_2_–5% CO_2_) at 37 °C and pH 7.3–7.5. We employed basal tensions of 5 mN (for tracheae) and 1 mN (for bronchi). Changes in muscle tension were recorded using PowerLab 4/30 software (version 7.3.7, AD Instruments) and expressed in milliNewtons (mN). Tissue viability was confirmed by assessing the contraction to a solution of 80 mM KCl, after which the preparations were extensively washed. Subsequently, the tissue contractility was assessed by obtaining cumulative concentration–response curves for the muscarinic agonist carbachol (0.001–10 µM; positive control), followed by extensive washing of the preparations with Krebs–Henseleit solution, after which venom was added to achieve final concentrations of 10, 30, and 100 µg/mL. After incubation for 10 min, the influence of the venom on tissue contractility was assessed by obtaining new concentration–response curves to carbachol. The parameters evaluated were the maximal contraction obtained (E_max_) and the concentration of agonist that induced 50% of the maximal response (EC_50_).

### 4.9. Quantification of Exhaled Nitric Oxide (NO)

Three groups of BALB/c mice (20–21 g) were used: Group 1 (control) received an i.v. injection of 100 µL of SS, Group 2 received an i.v. injection of 35 µg venom dissolved in 100 µL of SS, and Group 3 received an i.v. injection of 35 µg venom preincubated with 500 µM varespladib, as described above. Thirty minutes after venom injection, the mice were anesthetized (sodium thiopental, 70 mg/kg, i.p.) and tracheostomized. The levels of exhaled nitric oxide (eNO) were measured after a 10 min collection at the expiratory port of the ventilator using a Mylar Bag (Sievers Instruments Inc., Boulder, CO, USA). The concentration of NO was quantified by chemiluminescence (NO analyzer, model 280 NOA, Sievers Instruments Inc.) and was expressed in parts per billion (ppb).

### 4.10. Effect of Venom on Pulmonary Angiotensin-Converting Enzyme (ACE) Activity

Groups of BALB/c mice (20–21 g) received i.v. injections of 15 or 35 µg of venom, dissolved in 100 µL of SS. Controls received 100 µL of SS. After 40 min (mice injected with 35 µg) or 3 h (mice injected with 15 µg), the animals were euthanized with an overdose of isoflurane and exsanguinated; the thoracic cavity was opened and the lungs were removed, washed with SS, and immediately frozen in liquid nitrogen and stored at −80 °C until analysis. When required, pulmonary tissue was homogenized in a Polytron homogenizer at 4 °C in 40 mM sodium borate at pH 7.4 and using a ratio of 15 mL per gram of tissue. The homogenates were centrifuged (3000× *g*, 30 min, 4 °C) and the supernatants were collected and incubated with 0.05% Triton X-100 at 4 °C overnight. The samples were centrifuged again (10,000× *g*, 5 min, 4 °C) and the supernatant was assayed for angiotensin-converting enzyme activity as described by [81], based on [82], using N-hippuryl-L-histidyl-L-leucine (Sigma-Aldrich, St. Louis, MO, USA) as a substrate. This method is based on the quantification of hippuric acid released from the substrate by the enzyme (using a standard curve of this compound). Enzymatic activity was expressed as nmol hippuric acid/min/mg protein.

### 4.11. Analysis of Bronchoalveolar Lavage Fluid (BALF)

#### 4.11.1. Total Cell Counts in BALF

Groups of BALB/c mice (20–21 g) received an i.v. injection of 35 µg of venom dissolved in 100 µL of SS. A control group received 100 µL of SS. Mice were euthanized by isoflurane inhalation after 40 min and exsanguinated, and the trachea was exposed and cannulated with a 20 G catheter. Subsequently, 300 µL cold SS was flushed through the cannula and the liquid was collected. This process was repeated five times and approximately 1.5 mL of BALF was collected. The fluid was centrifuged (500× *g*, 4 °C, 10 min) and the pellet was resuspended in 200 µL of SS to perform cell counting. Total cell counts were obtained using a Neubauer chamber.

#### 4.11.2. Quantification of Total Protein and Hemoglobin in BALF

Groups of CD-1 mice (18–20 g) received an i.v. injection of 35 µg of venom, dissolved in 100 µL of SS. A control group received 100 µL of SS. BALF was obtained as described above. Mice were euthanized by isoflurane inhalation after 40 min and exsanguinated; the trachea was exposed and cannulated as described. Subsequently, 300 µL cold SS was flushed through the cannula and the liquid was collected. This process was repeated five times and approximately 1.5 mL of BALF were collected. After centrifugation (500× *g*, 4 °C, 10 min), the supernatants were separated and stored at −80 °C. Total protein concentration in the supernatants was determined by the bicinchoninic acid method (kit BCA1-1KT, Sigma-Aldrich) and the hemoglobin concentration was determined according to [83] using Drabkin reagent.

### 4.12. Effect of Anti-Inflammatory Drugs on the Venom-Induced Pulmonary Edema

To assess the role of various inflammatory mediators and the venom PLA_2_ activity, in venom-induced pulmonary edema in mice, the protein and hemoglobin concentrations in BALF were used as quantitative parameters to monitor pulmonary edema. To examine the involvement of PLA_2_ activity, venom was incubated with either SS (control) or varespladib (as previously described), and 100 µL aliquots containing 35 µg of venom were injected i.v. into groups of CD-1 mice (18–20 g). Forty minutes after injection, the mice were euthanized with isoflurane and BALF was collected and processed as described above for the quantification of total protein and hemoglobin concentrations.

The influence of the following inhibitors of inflammatory mediators on venom-induced edema was assessed: (a) L-NAME, an inhibitor of NO synthase, dissolved in SS, 50 mg/kg, i.p.; (b) indomethacin, an inhibitor of cyclooxygenase (COX), dissolved in 5% sodium bicarbonate solution, 10 mg/kg, i.p.; (c) HOE-140, a bradykinin receptor antagonist, dissolved in SS, 150 nmol/kg, i.p.; (d) pentoxifylline, an inhibitor of tumor necrosis factor-α (TNF-α), dissolved in SS, 3.5 mg/kg, i.p.; and (e) anti-neutrophil monoclonal antibody, 1 mg in 125 µL of SS per mouse, i.p. Control mice were injected with 100 µL of SS alone i.p. The first four inhibitors were administered 30 min before venom injection, whereas the anti-neutrophil antibody was administered 24 h before venom. Then, 30 min (or 24 h in the case of mice receiving anti-neutrophil antibody) after the administration of the inhibitors or SS, the mice received an i.v. injection of 35 µg of venom in 100 µL of SS. One group of mice that did not receive the drugs was treated with 35 µg of venom, dissolved in 100 µL SS, and another group received 100 µL SS. The mice were euthanized 40 min later with isoflurane, and BALF was collected as described above for the quantification of total protein and hemoglobin concentrations.

### 4.13. Statistical Analyses

Numerical data were expressed as the mean ± SD for the number of animals or experiments indicated. Statistical comparisons between two groups were performed using Student’s unpaired two-tailed *t*-test, and analysis of variance (ANOVA) was used when ≥3 experimental groups were compared, followed by post hoc comparisons using the Tukey–Kramer test or Dunnett’s test. *p* values < 0.05 indicated significance and all statistical analyses were performed using IBM^®^ SPSS Statistics software version 25.

## Data Availability

The original data presented in this study are included in the article/Supplementary Materials. Further inquiries can be directed to the corresponding author.

## Supplementary Materials

The following supporting information can be downloaded at: https://www.mdpi.com/article/10.3390/toxins17060302/s1, Figure S1: Total cell counts in bronchoalveolar lavage fluid (BALF) obtained from mice injected i.v. with either saline solution (SS, control), venom (35 µg) or venom (35 µg) preincubated with 500 µM varespladib (Var). The mice were injected with SS or venom and, after 40 min, were euthanized and exsanguinated. Subsequently, 300 µL of cold SS was flushed through the cannula and the liquid was collected. This process was repeated five times and approximately 1.5 mL of BAF lavage was recovered. After centrifugation, the pellet was resuspended in SS for cell counting. The columns represent the mean ± SD (n = 3 for controls and 4–5 for venom-injected mice). There were no significant differences among the treatments. (See Section 4.11 for full details); Table S1: Raw data of the experiments performed in this study and used to prepare the graphs included in the manuscript.

## References

[1] Gutiérrez J.M., Calvete J.J., Habib A.G., Harrison R.A., Williams D.J., Warrell D.A. (2017). Snakebite Envenoming. Nat. Rev. Dis. Primers.

[2] Warrell D.A. (2010). Snake Bite. Lancet.

[3] Gutiérrez J.M., Ownby C.L. (2003). Skeletal Muscle Degeneration Induced by Venom Phospholipases A_2_: Insights into the Mechanisms of Local and Systemic Myotoxicity. Toxicon.

[4] Harris J.B. (2003). Myotoxic Phospholipases A_2_ and the Regeneration of Skeletal Muscles. Toxicon.

[5] Hardy D., Besnard A., Latil M., Jouvion G., Briand D., Thépenier C., Pascal Q., Guguin A., Gayraud-Morel B., Cavaillon J.M. (2016). Comparative Study of Injury Models for Studying Muscle Regeneration in Mice. PLoS ONE.

[6] Teixeira C., Moreira V., Gutiérrez J.M. (2017). Venoms. Inflammation—From Molecular and Cellular Mechanisms to the Clinic.

[7] Benvenuti L.A., França F.O.S., Barbaro K.C., Nunes J.R., Cardoso J.L.C. (2003). Pulmonary Haemorrhage Causing Rapid Death after *Bothrops jararacussu* Snakebite: A Case Report. Toxicon.

[8] Palangasinghe D.R., Weerakkody R.M., Dalpatadu C.G., Gnanathasan C.A. (2015). A Fatal Outcome Due to Pulmonary Hemorrhage Following Russell’s Viper Bite. Saudi Med. J..

[9] Pinto L.J., Fernández L.L., Gutiérrez J.M., Simón D.S., Ceballos Z., Aguilar L.F., Sierra M. (2019). Case Report: Hemothorax in Envenomation by the Viperid Snake *Bothrops asper*. Am. J. Trop. Med. Hyg..

[10] Escalante T., Rucavado A., Fox J.W., Gutiérrez J.M. (2011). Key Events in Microvascular Damage Induced by Snake Venom Hemorrhagic Metalloproteinases. J. Proteom..

[11] White J. (2005). Snake Venoms and Coagulopathy. Toxicon.

[12] Silveira K.S.O., Boechem N.T., Do Nascimento S.M., Murakami Y.L.B., Barboza A.P.B., Melo P.A., Castro P., De Moraes V.L.G., Rocco P.R.M., Zin W.A. (2004). Pulmonary Mechanics and Lung Histology in Acute Lung Injury Induced by *Bothrops jararaca* Venom. Respir. Physiol. Neurobiol..

[13] Escalante T., Núñez J., Moura Da Silva A.M., Rucavado A., Theakston R.D.G., Gutiérrez J.M. (2003). Pulmonary Hemorrhage Induced by Jararhagin, a Metalloproteinase from *Bothrops jararaca* Snake Venom. Toxicol. Appl. Pharmacol..

[14] Castro A.C., Escalante T., Rucavado A., Gutiérrez J.M. (2021). Basement Membrane Degradation and Inflammation Play a Role in the Pulmonary Hemorrhage Induced by a P-III Snake Venom Metalloproteinase. Toxicon.

[15] Uma B., Veerabasappa Gowda T. (2000). Molecular Mechanism of Lung Hemorrhage Induction by VRV-PL-VIIIa from Russell’s Viper (*Vipera russelli*) Venom. Toxicon.

[16] Wei X.L., Wei J.F., Li T., Qiao L.Y., Liu Y.L., Huang T., He S.H. (2007). Purification, Characterization and Potent Lung Lesion Activity of an L-Amino Acid Oxidase from *Agkistrodon blomhoffii ussurensis* Snake Venom. Toxicon.

[17] Nonaka P.N., Amorim C.F., Paneque Peres A.C., Silva C.A.M., Zamuner S.R., Ribeiro W., Cogo J.C., Vieira R.P., Dolhnikoff M., de Oliveira L.V.F. (2008). Pulmonary Mechanic and Lung Histology Injury Induced by *Crotalus durissus terrificus* Snake Venom. Toxicon.

[18] Ali S.A., Alam J.M., Abbasi A., Zaidi Z.H., Stoeva S., Voelter W. (2000). Sea Snake *Hydrophis cyanocinctus* Venom. II. Histopathological Changes, Induced by a Myotoxic Phospholipase A_2_ (PLA_2_-H1). Toxicon.

[19] Cher C.D.N., Armugam A., Lachumanan R., Coghlan M.W., Jeyaseelan K. (2003). Pulmonary Inflammation and Edema Induced by Phospholipase A_2_: Global Gene Analysis and Effects on Aquaporins and Na^+^/K^+^-ATPase. J. Biol. Chem..

[20] Malaquin S., Bayat S., Arab O., Mourier G., Lorne E., Kamel S., Dupont H., Ducancel F., Mahjoub Y. (2016). Respiratory Effects of Sarafotoxins from the Venom of Different Atractaspis Genus Snake Species. Toxins.

[21] Pla D., Bande B.W., Welton R.E., Paiva O.K., Sanz L., Segura Á., Wright C.E., Calvete J.J., Gutiérrez J.M., Williams D.J. (2017). Proteomics and Antivenomics of Papuan Black Snake (*Pseudechis papuanus*) Venom with Analysis of Its Toxicological Profile and the Preclinical Efficacy of Australian Antivenoms. J. Proteom..

[22] Goldenberg J., Cipriani V., Jackson T.N.W., Arbuckle K., Debono J., Dashevsky D., Panagides N., Ikonomopoulou M.P., Koludarov I., Li B. (2018). Proteomic and Functional Variation within Black Snake Venoms (Elapidae: Pseudechis). Comp. Biochem. Physiol. C Toxicol. Pharmacol..

[23] Laing G.D., Kamiguti A.S., Wilkinson M.C., Lowe G.M., Theakston R.D.G. (1995). Characterisation of a Purified Phospholipase A_2_ from the Venom of the Papuan Black Snake (*Pseudechis papuanus*). Biochim. Biophys. Acta.

[24] Lomonte B., Križaj I. (2021). Snake Venom Phospholipase A_2_ Toxins. Handbook of Venoms and Toxins of Reptiles.

[25] Bos L.D.J., Ware L.B. (2022). Acute Respiratory Distress Syndrome: Causes, Pathophysiology, and Phenotypes. Lancet.

[26] Kitsiouli E., Nakos G., Lekka M.E. (2009). Phospholipase A_2_ Subclasses in Acute Respiratory Distress Syndrome. Biochim. Biophys. Acta.

[27] Letsiou E., Htwe Y.M., Dudek S.M. (2021). Secretory Phospholipase A_2_ Enzymes in Acute Lung Injury. Cell Biochem. Biophys..

[28] Seeds M.C., Grier B.L., Suckling B.N., Safta A.M., Long D.L., Waite B.M., Morris P.E., Hite R.D. (2012). Secretory Phospholipase A_2_ Mediated Depletion of Phosphatidylglycerol in Early Acute Respiratory Distress Syndrome. Am. J. Med. Sci..

[29] Kitsiouli E., Tenopoulou M., Papadopoulos S., Lekka M.E. (2021). Phospholipases A_2_ as Biomarkers in Acute Respiratory Distress Syndrome. Biomed. J..

[30] Aeffner F., Bolon B., Davis I.C. (2015). Mouse Models of Acute Respiratory Distress Syndrome. Toxicol. Pathol..

[31] Bastarache J.A., Blackwell T.S. (2009). Development of Animal Models for the Acute Respiratory Distress Syndrome. DMM Dis. Models Mech..

[32] Rucavado A., Escalante T., Gutiérrez J.M. (2004). Effect of the Metalloproteinase Inhibitor Batimastat in the Systemic Toxicity Induced by *Bothrops asper* Snake Venom: Understanding the Role of Metalloproteinases in Envenomation. Toxicon.

[33] Silva A., Gunawardena P., Weilgama D., Maduwage K., Gawarammana I. (2012). Comparative In-Vivo Toxicity of Venoms from South Asian Hump-Nosed Pit Vipers (Viperidae: Crotalinae: *Hypnale*). BMC Res. Notes.

[34] Herrera C., Rucavado A., Warrell D.A., Gutiérrez J.M. (2013). Systemic Effects Induced by the Venom of the Snake *Bothrops caribbaeus* in a Murine Model. Toxicon.

[35] Bahloul M., Chaari A., Dammak H., Samet M., Chtara K., Chelly H., Ben Hamida C., Kallel H., Bouaziz M. (2013). Pulmonary Edema Following Scorpion Envenomation: Mechanisms, Clinical Manifestations, Diagnosis and Treatment. Int. J. Cardiol..

[36] Isbister G.K., Bawaskar H.S. (2014). Scorpion Envenomation. N. Engl. J. Med..

[37] Deshpande S.B., Akella A. (2012). Non-Cardiogenic Mechanisms for the Pulmonary Edema Induced by Scorpion Venom. Int. J. Cardiol..

[38] França F.O., Benvenuti L.A., Fan H.W., Dos Santos D.R., Hain S.H., Picchi-Martins F.R., Cardoso J.L., Kamiguti A.S., Theakston R.D., Warrell D.A. (1994). Severe and Fatal Mass Attacks by “Killer” Bees (Africanized Honey Bees—*Apis mellifera scutellata*) in Brazil: Clinicopathological Studies with Measurement of Serum Venom Concentrations. Q. J. Med..

[39] Cavalcante J.S., Riciopo P.M., Pereira A.F.M., Jeronimo B.C., Angstmam D.G., Pôssas F.C., de Andrade Filho A., Cerni F.A., Pucca M.B., Ferreira Junior R.S. (2024). Clinical Complications in Envenoming by Apis Honeybee Stings: Insights into Mechanisms, Diagnosis, and Pharmacological Interventions. Front. Immunol..

[40] Thumtecho S., Suteparuk S., Sitprija V. (2023). Pulmonary Involvement from Animal Toxins: The Cellular Mechanisms. J. Venom. Anim. Toxins Incl. Trop. Dis..

[41] Lewin M., Samuel S., Merkel J., Bickler P. (2016). Varespladib (LY315920) Appears to Be a Potent, Broad-Spectrum, Inhibitor of Snake Venom Phospholipase A_2_ and a Possible Pre-Referral Treatment for Envenomation. Toxins.

[42] Lewin M.R., Carter R.W., Matteo I.A., Samuel S.P., Rao S., Fry B.G., Bickler P.E. (2022). Varespladib in the Treatment of Snakebite Envenoming: Development History and Preclinical Evidence Supporting Advancement to Clinical Trials in Patients Bitten by Venomous Snakes. Toxins.

[43] Bazan-Socha S., Zuk J., Plutecka H., Jakiela B., Mlicka-Kowalczyk E., Krzyzanowski B., Marcinkiewicz C., Zareba L., Bazan J.G., Musial J. (2014). Blocking of A1β1 and A2β1 Adhesion Molecules Inhibits Eosinophil Migration through Human Lung Microvascular Endothelial Cell Monolayer. Postepy. Hig. Med. Dosw..

[44] Komori Y., Murakami E., Uchiya K.I., Nonogaki T., Nikai T. (2014). Okinalysin, a Novel P-I Metalloproteinase from *Ovophis okinavensis*: Biological Properties and Effect on Vascular Endothelial Cells. Toxins.

[45] Suzuki K., Nakamura M., Hatanaka Y., Kayanoki Y., Tatsumi H., Taniguchi N. (1997). Induction of Apoptotic Cell Death in Human Endothelial Cells Treated with Snake Venom: Implication of Intracellular Reactive Oxygen Species and Protective Effects of Glutathione and Superoxide Dismutases. J. Biochem..

[46] Gallagher P.G., Bao Y., Serrano S.M.T., Kamiguti A.S., Theakston R.D.G., Fox J.W. (2003). Use of Microarrays for Investigating the Subtoxic Effects of Snake Venoms: Insights into Venom-Induced Apoptosis in Human Umbilical Vein Endothelial Cells. Toxicon.

[47] Albrecht E.A., Dhanasekaran S.M., Tomlins S. (2011). Immediate Early Inflammatory Gene Responses of Human Umbilical Vein Endothelial Cells to Hemorrhagic Venom. Inflamm. Res..

[48] Machado A.R.T., Aissa A.F., Ribeiro D.L., Costa T.R., Ferreira R.S., Sampaio S.V., Antunes L.M.G. (2019). Cytotoxic, Genotoxic, and Oxidative Stress-Inducing Effect of an L-Amino Acid Oxidase Isolated from *Bothrops jararacussu* Venom in a Co-Culture Model of HepG2 and HUVEC Cells. Int. J. Biol. Macromol..

[49] Salazar E., Cirilo A., Reyes A., Barrientos M., Galan J., Sánchez E.E., Suntravat M. (2024). Snake Venom Cysteine-Rich Secretory Protein from Mojave Rattlesnake Venom (Css-CRiSP) Induces Acute Inflammatory Responses on Different Experimental Models. Toxicon X.

[50] Bittenbinder M.A., Bonanini F., Kurek D., Vulto P., Kool J., Vonk F.J. (2024). Using Organ-on-a-Chip Technology to Study Haemorrhagic Activities of Snake Venoms on Endothelial Tubules. Sci. Rep..

[51] Oliveira V.Q., Santos L.C., Teixeira S.C., Correia T.M.L., Andrade L.O.S.B., Gimenes S.N.C., Colombini M., Marques L.M., Jiménez-Charris E., Freitas-de-Sousa L.A. (2024). Antiangiogenic Properties of BthMP, a P–I Metalloproteinase from *Bothrops moojeni* Snake Venom by VEGF Pathway in Endothelial Cells. Biochem. Biophys. Res. Commun..

[52] Chen L., Liu C., Chang C. (1994). Isolation and Characterization of a Toxic Phospholipase A_2_ from the Venom of the Taiwan Habu (*Trimeresurus mucrosquamatus*). Biotechnol. Appl. Biochem..

[53] de Albuquerque Modesto J.C., Spencer P.J., Fritzen M., Valença R.C., Oliva M.L.V., da Silva M.B., Chudzinski-Tavassi A.M., Guarnieri M.C. (2006). BE-I-PLA_2_, a Novel Acidic Phospholipase A_2_ from *Bothrops erythromelas* Venom: Isolation, Cloning and Characterization as Potent Anti-Platelet and Inductor of Prostaglandin I_2_ Release by Endothelial Cells. Biochem. Pharmacol..

[54] Conlon J.M., Attoub S., Arafat H., Mechkarska M., Casewell N.R., Harrison R.A., Calvete J.J. (2013). Cytotoxic Activities of [Ser^49^]Phospholipase A_2_ from the Venom of the Saw-Scaled Vipers *Echis ocellatus*, *Echis pyramidum Leakeyi*, *Echis carinatus sochureki*, and *Echis coloratus*. Toxicon.

[55] Polloni L., Azevedo F.V.P.V., Teixeira S.C., Moura E., Costa T.R., Gimenes S.N.C., Correia L.I.V., Freitas V., Yoneyama K.A.G., Rodrigues R.S. (2021). Antiangiogenic Effects of Phospholipase A_2_ Lys49 BnSP-7 from *Bothrops pauloensis* Snake Venom on Endothelial Cells: An in Vitro and Ex Vivo Approach. Toxicol. In Vitro.

[56] Santos L.C., Oliveira V.Q., Teixeira S.C., Correia T.M.L., Andrade L.O.S.B., Polloni L., Marques L.M., Clissa P.B., Baldo C., Ferro E.A.V. (2024). PLA_2_-MjTX-II from *Bothrops moojeni* Snake Venom Exhibits Antimetastatic and Antiangiogenic Effects on Human Lung Cancer Cells. Toxicon.

[57] de Melo Cordeiro Eulálio M., de Lima A.M., Brant R.S.C., Francisco A.F., Santana H.M., Paloschi M.V., da Silva Setúbal S., da Silva C.P., Silva M.D.S., Boeno C.N. (2025). Characterization of a Novel Acidic Phospholipase A_2_ Isolated from the Venom of *Bothrops mattogrossensis*: From Purification to Structural Modeling. Int. J. Biol. Macromol..

[58] Ji J., Sun L., Luo Z., Zhang Y., Xianzheng W., Liao Y., Tong X., Shan J. (2021). Potential Therapeutic Applications of Pulmonary Surfactant Lipids in the Host Defence Against Respiratory Viral Infections. Front. Immunol..

[59] Autilio C., Pérez-Gil J. (2019). Understanding the Principle Biophysics Concepts of Pulmonary Surfactant in Health and Disease. Arch. Dis. Child. Fetal Neonatal Ed..

[60] Mok Y.H., Lee J.H., Rehder K.J., Turner D.A. (2014). Adjunctive Treatments in Pediatric Acute Respiratory Distress Syndrome. Expert Rev. Respir. Med..

[61] Kamiguti A.S., Laing G.D., Lowe G.M., Zuzel M., Warrell D.A., Theakston R.D.G. (1994). Biological Properties of the Venom of the Papuan Black Snake (*Pseudechis papuanus*): Presence of a Phospholipase A_2_ Platelet Inhibitor. Toxicon.

[62] Lalloo D., Trevett A., Black J., Mapao J., Naraqi S., Owens D., Hutton R., Theakston R.D.G., Warrell D.A. (1994). Neurotoxicity and Haemostatic Disturbances in Patients Envenomed by the Papuan Black Snake (*Pseudechis papuanus*). Toxicon.

[63] Ramasamy S., Fry B.G., Hodgson W.C. (2005). Neurotoxic Effects of Venoms from Seven Species of Australasian Black Snakes (Pseudechis): Efficacy of Black and Tiger Snake Antivenoms. Clin. Exp. Pharmacol. Physiol..

[64] Kuruppu S., Reeve S., Smith A.I., Hodgson W.C. (2005). Isolation and Pharmacological Characterisation of Papuantoxin-1, a Postsynaptic Neurotoxin from the Venom of the Papuan Black Snake (*Pseudechis papuanus*). Biochem. Pharmacol..

[65] Bastarache J.A., Roberts L.J., Ware L.B. (2014). Thinking Outside the Cell: How Cell-Free Hemoglobin Can Potentiate Acute Lung Injury. Am. J. Physiol. Lung Cell. Mol. Physiol..

[66] Janz D.R., Ware L.B. (2015). The Role of Red Blood Cells and Cell-Free Hemoglobin in the Pathogenesis of ARDS. J. Intensive Care.

[67] Meegan J.E., Bastarache J.A., Ware L.B. (2021). Toxic Effects of Cell-Free Hemoglobin on the Microvascular Endothelium: Implications for Pulmonary and Nonpulmonary Organ Dysfunction. Am. J. Physiol. Lung Cell. Mol. Physiol..

[68] Meduri G.U., Kohler G., Headley S., Tolley E., Stentz F., Postlethwaite A. (1995). Inflammatory Cytokines in the BAL of Patients with ARDS. Persistent Elevation over Time Predicts Poor Outcome. Chest.

[69] Grommes J., Soehnlein O. (2011). Contribution of Neutrophils to Acute Lung Injury. Mol. Med..

[70] Butt Y., Kurdowska A., Allen T.C. (2016). Acute Lung Injury: A Clinical and Molecular Review. Arch. Pathol. Lab. Med..

[71] Rex D.A.B., Vaid N., Deepak K., Dagamajalu S., Prasad T.S.K. (2022). A Comprehensive Review on Current Understanding of Bradykinin in COVID-19 and Inflammatory Diseases. Mol. Biol. Rep..

[72] Brett S.J., Evans T.W. (1998). Measurement of Endogenous Nitric Oxide in the Lungs of Patients with the Acute Respiratory Distress Syndrome. Am. J. Respir. Crit. Care Med..

[73] Radi R. (2018). Oxygen Radicals, Nitric Oxide, and Peroxynitrite: Redox Pathways in Molecular Medicine. Proc. Natl. Acad. Sci. USA.

[74] Baron R.M., Carvajal I.M., Fredenburgh L.E., Liu X., Porrata Y., Cullivan M.L., Haley K.J., Sonna L.A., De Sanctis G.T., Ingenito E.P. (2004). Nitric Oxide Synthase-2 down-Regulates Surfactant Protein-B Expression and Enhances Endotoxin-Induced Lung Injury in Mice. FASEB J..

[75] Mikawa K., Nishina K., Takao Y., Obara H. (2003). ONO-1714, a Nitric Oxide Synthase Inhibitor, Attenuates Endotoxin-Induced Acute Lung Injury in Rabbits. Anesth. Analg..

[76] Lin H.I., Chu S.J., Wang D., Chen H.I., Hsu K. (2003). Effects of an Endogenous Nitric Oxide Synthase Inhibitor on Phorbol Myristate Acetate-Induced Acute Lung Injury in Rats. Clin. Exp. Pharmacol. Physiol..

[77] Taddei S., Bortolotto L. (2016). Unraveling the Pivotal Role of Bradykinin in ACE Inhibitor Activity. Am. J. Cardiovasc. Drugs.

[78] Holzer M., Mackessy S.P. (1996). An Aqueous Endpoint Assay of Snake Venom Phospholipase A_2_. Toxicon.

[79] Hantos Z., Adamicza A., Govaerts E., Daroczy B. (1992). Mechanical Impedances of Lungs and Chest Wall in the Cat. J. Appl. Physiol..

[80] Lourenço J.D., Neves L.P., Olivo C.R., Duran A., Almeida F.M., Arantes P.M.M., Prado C.M., Leick E.A., Tanaka A.S., Martins M.A. (2014). A Treatment with a Protease Inhibitor Recombinant from the Cattle Tick (*Rhipicephalus boophilus microplus*) Ameliorates Emphysema in Mice. PLoS ONE.

[81] Hurst P.L., Lovell-Smith C.J. (1981). Optimized Assay for Serum Angiotensin-Converting Enzyme Activity. Clin. Chem..

[82] Cushman D.W., Cheung H.S. (1971). Spectrophotometric Assay and Properties of the Angiotensin-Converting Enzyme of Rabbit Lung. Biochem. Pharmacol..

[83] van Kampen E.J., Zijlstra W.G. (1965). Determination of Hemoglobin and Its Derivatives. Adv. Clin. Chem..

