# Supplementary figures and images for: An Experimental Model of Acute Pulmonary Damage Induced by the Phospholipase A2-Rich Venom of the Snake Pseudechis papuanus
Source: Toxins (Basel). 2025 Jun 12;17(6):302. doi: 10.3390/toxins17060302 (PMC12197351; doi:10.3390/toxins17060302)

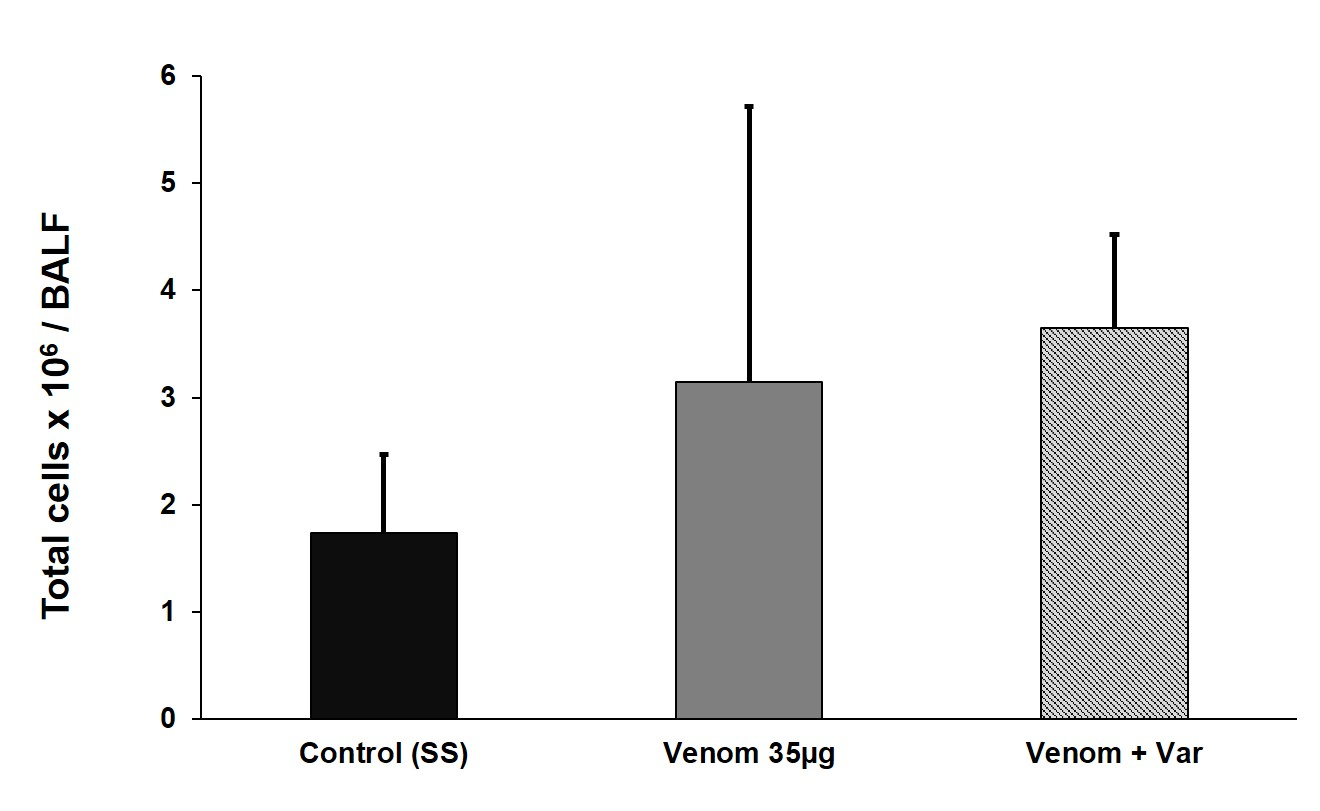

Supplement: Supplementary file 1 [file toxins-17-00302-s001.zip › Supplementary figure S1.tiff]
